# Carnosine Inhibits the Proliferation of Human Gastric Cancer SGC-7901 Cells through Both of the Mitochondrial Respiration and Glycolysis Pathways

**DOI:** 10.1371/journal.pone.0104632

**Published:** 2014-08-12

**Authors:** Yao Shen, Jianbo Yang, Juan Li, Xiaojie Shi, Li Ouyang, Yueyang Tian, Jianxin Lu

**Affiliations:** Key Laboratory of Laboratory Medicine, Ministry of Education, Attardi Institute of Mitochondrial Biomedicine and Zhejiang Provincial Key Laboratory of Medical Genetics, College of Laboratory Medicine and Life sciences, Wenzhou Medical University, Wenzhou, China; Duke University Medical Center, United States of America

## Abstract

Carnosine, a naturally occurring dipeptide, has been recently demonstrated to possess anti-tumor activity. However, its underlying mechanism is unclear. In this study, we investigated the effect and mechanism of carnosine on the cell viability and proliferation of the cultured human gastric cancer SGC-7901 cells. Carnosine treatment did not induce cell apoptosis or necrosis, but reduced the proliferative capacity of SGC-7901 cells. Seahorse analysis showed SGC-7901 cells cultured with pyruvate have active mitochondria, and depend on mitochondrial oxidative phosphorylation more than glycolysis pathway for generation of ATP. Carnosine markedly decreased the absolute value of mitochondrial ATP-linked respiration, and reduced the maximal oxygen consumption and spare respiratory capacity, which may reduce mitochondrial function correlated with proliferative potential. Simultaneously, carnosine also reduced the extracellular acidification rate and glycolysis of SGC-7901 cells. Our results suggested that carnosine is a potential regulator of energy metabolism of SGC-7901 cells both in the anaerobic and aerobic pathways, and provided a clue for preclinical and clinical evaluation of carnosine for gastric cancer therapy.

## Introduction

Gastric cancer is one of the most common malignancies in the world. In the economically developing countries, gastric cancer is the second cause of cancer-related death [1], [2]. In spite of the improvement in surgical and multimodal therapy, the overall 5-year survival rate is still low (15% to 35%) because of the high recurrence rates, invasion and metastasis [3]. Therefore, in the present, to discover more effective anti-tumor drugs with fewer side effects is needed.

L-Carnosine (β-alanyl-L-histidine) is a naturally occurring dipeptide that is synthesized by endogenous carnosine synthetase. It is widely distributed in mammalian brain, skeletal muscle, stomach, kidneys, heart and skin [4], [5]. So far, not much is known about its physiological function but several putative roles have been considered, such as neurotransmitter, anti-inflammatory agent, free radical scavenger, mobile organic pH buffer and metal chelator [6], [7]. It has been reported that carnosine is a potential therapeutic agent for the treatment of Alzheimer’s disease, stroke, diabetes, and other diseases of the sense organs [8], [9]. Just recently, it was demonstrated that carnosine may also have an anti-tumorigenic effects. For example, carnosine has been reported to possess the ability to inhibit malignant gliomas growth [10], and this effect may be mediated by an influence on glycolytic energy metabolism, the best characterized metabolic phenotype observed in tumour cells, known as Warburg effect [11], [12].

Recently, the importance of mitochondria as oxygen sensors as well as producers of ATP has become a focal point of cancer research, and studies have showed an important phenomenon that mitochondrial metabolism, particularly citric acid cycle activity is important for the rapid proliferation of multiple cancer cell types [13], [14]. However, in the case of human gastric cancer cells, little information is available to what extent glycolysis and mitochondrial oxidative phosphorylation (OXPHOS) contribute to the cellular energy production and rapid proliferation. Whether carnosine can also inhibit the growth of human gastric cancer cells remains unknown. And whether the inhibitive effect of carnosine on tumor cells growth is also related to its action on mitochondrial respiration and OXPHOS remains unclear.

Recently, the Seahorse Bioscience XF96 Extracellular Flux Analyzer has been used to simultaneously and continually monitor both the aerobic and glycolytic components of cellular bioenergetics [15]. Therefore, in the present study, we explored the effects of carnosine on the growth of human gastric cancer cells and to further characterize the bioenergetic profile of cultured human gastric cancer cell SGC-7901 and the roles of carnosine in SGC-7901 cells energy metabolism with the Seahorse Bioscience XF96 Extracellular Flux Analyzer and other related technologies.

## Materials and Methods

### Reagents

L-Carnosine, sodium pyruvate, rotenone, carbonyl cyanide p-trifluoromethoxyphenyl-hydrazone (FCCP), antimycin A, 3-[4,5-dimethylthiazol-2-yl]-2,5-diphenyltetra-zolium bromide (MTT), methanol, lactic acid were from Sigma (St. Louis, MO, USA). Penicillin, streptomycin, L-glutamine, trypsin, Dulbecco’s modified Eagle’s medium (DMEM), fetal bovine serum were from GIBCO-BRL (Grand Island, NY, USA). Annexin V-FITC/PI apoptosis detection kit, BCA Protein Assay Kit and ATP Assay Kit were bought from Beyotime Institute of Biotechnology ((Nanjing, China). XF assay medium and XF calibrant solution were bought from Seahorse Bioscience.

### Cell culture

Human gastric cancer cell line SGC-7901 (SGC-7901), human liver hepatocellular carcinoma cell line (HepG2) and rat C6 glioma cell line (C6) were purchased from the Shanghai Institute cell bank, Chinese Academy of Science (the original source is American Type Culture Collection, ATCC, Manassas, VA, USA). Cells were cultured in DMEM medium supplemented with 10% fetal bovine serum (FBS), 100 U/ml penicillin G, and 100 µg/ml streptomycin, and maintained at 37°C and 5% CO_2_ in a humidified incubator. Cells were trypsinized at a ratio of 1∶3 after confluence using 0.25% trypsin. Subcultured cells were seeded onto 96-, 24- or 6-well plates at densities of 2×10^3^, 5×10^4^, 2×10^5^ or 1×10^6^ cells/well, respectively.

### MTT reduction assay

Cell metabolic activity was monitored by the colorimetric MTT assay as described previously [16]. Briefly, cells were cultured on 96-well plates and there were 6 wells in each group. At the end of experiments, the cells were incubated with 0.5 mg/ml MTT for 4 h at 37°C. Then, the supernatant layer was removed, and 100 µL of dimethyl sulfoxide was added into each well. MTT metabolism was quantitated spectrophotometrically at 570 nm in a Biorad microplate reader. Results were expressed as the percentage of MTT reduction, taking the absorbance of control cells as 100%.

### Colony formation assay

Cells were plated in six-well plates at density of 100–200 cells per well, and then were treated with carnosine (20 mM). Clones were allowed to grow for 14 days in DMEM culture medium supplemented with 10% FBS, 100 U/ml penicillin G, and 100 µg/ml streptomycin. Cells were subsequently fixed with 70% ethanol and stained with Coomassie Brilliant Blue for analysis of colony formation as described previously [17].

### Flow Cytometric Assay of cell death

Cell death was quantified by Annexin V-FITC-PI (propidium iodide) double staining, using an Annexin V-FITC apoptosis detection kit according to the manufacturer’s suggestion. Briefly, cells were seeded in 6-well plates in DMEM medium. Cells were treated with 20 mM carnosine for 48 h, and then were collected and washed twice in ice-cold PBS, resuspended in binding buffer at a density of 1×10^6^ cells/ml. Cells were incubated simultaneously with fluorescein-labeled Annexin V and PI for 20 min and analyzed by flow cytometry. Annexin V-FITC generated signals were detected with an FITC signal detector (FL1, 525 nm). PI signals were monitored using a detector reserved for phycoerythrin emission (FL2, 575 nm). Data were analyzed using Cell Quest software from BD.

### Extracellular Flux Technology

To measure the oxygen consumption rate (OCR) and extracellular acidification rate (ECAR) of cells in different conditions, a Seahorse XF96 Extracellular Flux Analyzer (Seahorse Bioscience, Billerica, MA, USA) was used. This instrument allows for the sensitive measurement of glycolysis and multiple parameters of mitochondrial function, including basal OCR, spare respiratory capacity, maximal OCR, ATP-linked respiration and proton leak from adherent intact cultured cells. After baseline measurements, OCR and ECAR were measured after sequentially adding to each well 20 µl of oligomycin, FCCP and rotenone, to reach working concentrations of 1 µg/ml, 1 µM and 1 µM, respectively. All assays were conducted using a seeding density of 10000 cells/well in 200 µL of DMEM in a XF96 cell culture microplate (Seahorse Bioscience). The cells were switched to unbuffered DMEM supplemented with 2 mM sodium pyruvate and 20 mM carnosine 1 h prior to the beginning of the assay and maintained at 37°C. OCR is reported in the unit of picomoles per minute and ECAR is reported in milli-pH units (mpH) per minute.

### Determination of ATP Production

The ATP assay was performed according to the manufacturer’s instruction. Briefly, harvested cultured cells were lysed with a lysis buffer, followed by centrifugation at 10,000×g for 2 min, at 4°C. Finally, in 6-well plates, the level of ATP was determined by mixing 20 µl of the supernatant with 100 µl of luciferase reagent, which catalyzed the light production from ATP and luciferin. Luminance was measured by a monochromator microplate reader. Standard curve was also generated and the protein concentration of each group was determined using the BCA protein assay kit. Total ATP levels were expressed as nmol/mg protein.

### HPLC analysis of extracellular lactic acid

The concentration of lactic acid in the cell-free supernatant was measured by high-performance liquid chromatography (HPLC) combined with an ultraviolet detector using the technique as described previously. In brief, the prepared analysates were separated on Ecosil C-18 reversed column (3 µm, 250 mm×4.6 mm) using solvent A and solvent B [0.1 mol/l NH_4_H_2_PO_4_ (pH 3.4) diluted v/v in methanol 97∶3] with a flow rate of 0.5 ml/min. The temperature of the column was maintained at 25°C, and the wavelength of UV detection was set at 210 nm.

### Mitochondrial membrane potential assessment

The changes in relative mitochondrial membrane potential (Δ*Ψ*m) were assessed using the lipophilic cationic probe JC-1 5,5′,6,6′-tetrachloro-1,1′,3,3′-tetraethyl-benzamid azolocarbocyanine iodide (JC-1; Molecular Probes). The dye JC-1 undergoes a reversible change in fluorescence emission from green to greenish orange as Δ*Ψ*m increases. Cells with high Δ*Ψ*m form JC-1 aggregates and fluoresce red; those with low Δ*Ψ*m contain monomeric JC-1 and fluoresce green. After treatment with carnosine for 48 h, the culture medium was removed and the cells, grown on coverslips, were incubated in the dark with JC-1 at a final concentration of 2 µM for 20 min. The cells were rinsed with PBS twice and excited at 488 nm with an Olympus BX-51 fluorescence microscope.

### MtDNA copy number measurement

Absolute mtDNA copy number was measured by comparing PCR amplification of a mitochondria amplicon [human, NADH-ubiquinone oxidoreductase chain 4 (ND4)] with a nuclear amplicon (human, β-actin) from DNA isolated using a Qiagen DNA mini kit. Primer sequences are as follow: human β-actin (forward: 5′-ACCCACACTGTGCC CATCTAC-3′; reverse: 5′-TCGGTGAGGATCTTCATGAGGTA-3′); human ND4 (forward: 5′-TCCTCCTATCCCTCAACCCC-3′; reverse: 5′-CACAATCTGATGTTTT GGTTAAAC-3′). DNA templates were made of regions spanning each amplicon by PCR amplification. Dilutions from 1×10^8^ down to 1×10^2^ copies of the isolated DNA were used to generate a standard curve for quantification. The PCR cycling conditions consisted of an activation step at 95°C for 30 sec, followed by 40 cycles for 5 sec at 95°C and 30 sec at 58°C. All PCRs were performed on a Bio-Rad CFX Manager Real-Time PCR System (Applied Biosciences).

### Statistical Analysis

All data represent three or more independent experiments. Data were expressed as mean ± SD. Statistical analyses were conducted by SPSS 11.5 for Windows. One-way ANOVA (analysis of variance) followed by LSD (least significant difference) or Dunnett’s T3 *post-hoc* test (where equal variances were not assumed) was applied for multiple comparisons, whereas Student’s t-test was used for comparisons between two groups. *P*<0.05 was considered statistically significant.

## Results

### Effect of carnosine on SGC-7901 cells viability

**Figure 1:**
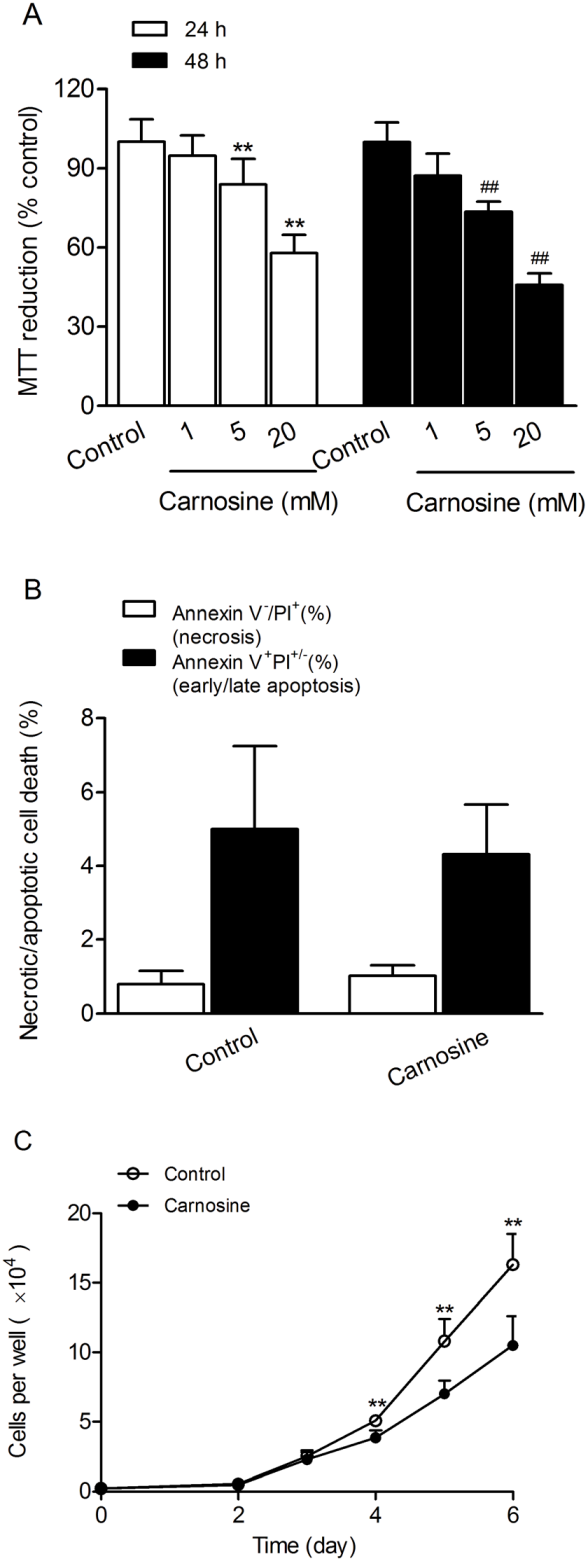
Effects of carnosine on SGC-7901 cell viability and proliferation. (A) Cells were pre-treated with different concentrations of carnosine for 24 or 48 h, and then the cell viability was assayed using the MTT reduction assay. Results were expressed as percentage of control, and were showed mean ± SD. n = 10–12. **P<0.01 vs. control in 24 h group; ^##^ *P*<0.01 vs. control in 48 h group. (B) Cells were treated with 20 mM carnosine for 48 h, and then cell death was determined by PI and annexin V-FITC staining followed by flow cytometry. (C) SGC-7901 cells were treated with 20 mM carnosine and the total cell number was calculated after carnosine treatment for 2, 3, 4, 5, 6 days using cell counting plate. Data were expressed as mean ± SD. n = 6. **P<0.01 vs. control.

To determine the effect of carnosine on human gastric cancer SGC-7901 cells viability, MTT reduction assay was used. Results showed that carnosine treatment significantly reduced cell viability in a time- and concentration-dependent manner. Carnosine at concentrations of 5 and 20 mM markedly reduced cell viability to 84.0% and 57.9% of control at 24 h, and to 73.5% and 45.9% of control at 48 h, respectively (Fig. 1A). However, carnosine at concentration of 1 mM did not affect SGC-7901 cells viability at 24 or 48 h. We further used flow cytometry to assay whether carnosine could cause SGC-7901 cell necrosis or apoptosis. Surprisingly, the results showed that carnosine treatment for 48 h did not induce necrotic or apoptotic cell death in SGC-7901 cells (Fig. 1B). Because MTT reduction is also interpreted to be indicative of cellular metabolic activity, and the MTT value of a cell population is determined by both the number of viable cells present and their relative metabolic rates, so we next to calculate the cell number in a parallel experiment with identically treated SGC-7901 cells using cell counting plate. We found that the cell number in carnosine treated for 48 h group was similar to that in control group (Fig. 1C), thus indicating that the reduced cell viability induced by carnosine treatment for 48 h in SGC-7901 cells was due to metabolic changes but not due to cell death or cell proliferation.

To verify whether these actions of carnosine also exist in other cancer cells, HepG2 and C6 cells were used. The results showed that 20 mM carnosine treatment for 48 h did not induce cell death (Table. S1) or proliferation, but markedly reduced MTT reducing activity both in HepG2 and C6 cells (Fig. S1).

### Choronic treatment with carnosine inhibited SGC-7901 cells colonies formation

**Figure 2:**
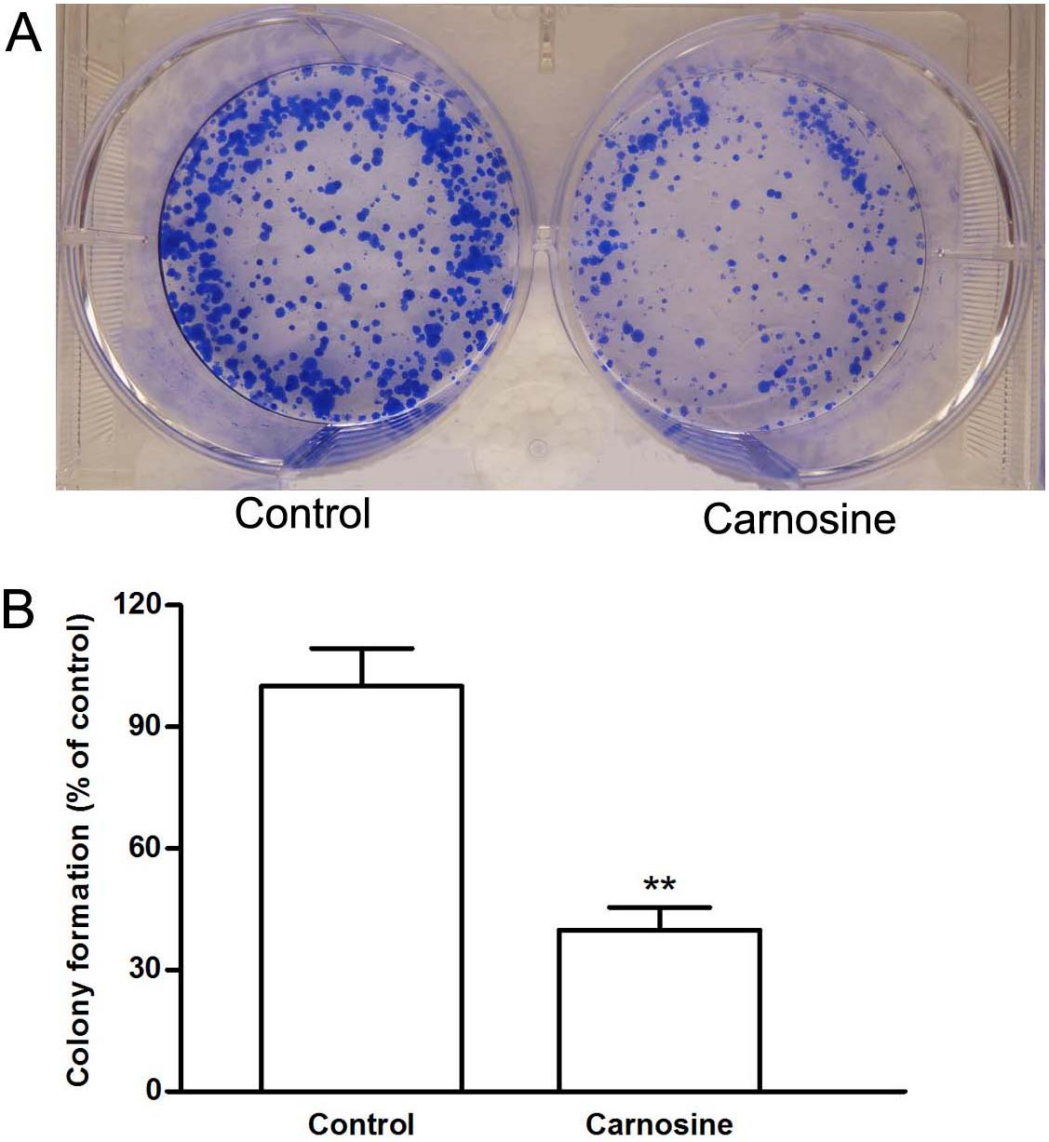
Effect of carnosine on SGC-7901 cells colony formation. (A) Representative images of the cloning wells. Cells were seeded at low density in DMEM supplement with or without carnosine (20 mM) for 14 days. The colonies were subsequently fixed with 70% ethanol and stained with Coomassie Brilliant Blue for analysis of colony formation. (B) Quantitative image analysis of colonies in cultured SGC7901 cells. Data were expressed as mean ± SD. n = 6. **P<0.01 vs. control group.

To examine whether choronic exposure to carnosine could affect the proliferative capacity of SGC-7901 cells, the cells were seeded at a low density (100–200 cells/well) and allowed to form colonies for 14 days in DMEM supplemented with 20 mM carnosine. As shown in Fig. 2, choronic exposure to carnosine reduced colonies formation to 39.9% of control.

### Bioenergetic characterization of cultured SGC-7901 cells

**Figure 3:**
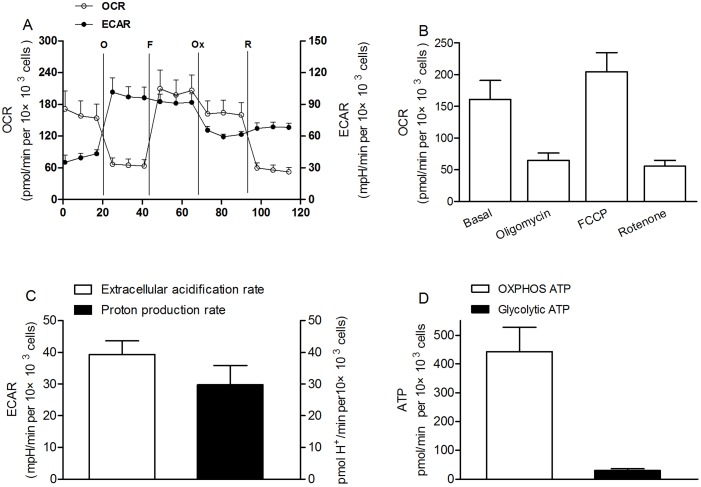
Bioenergetic characterization of SGC-7901 cells cultured in DMEM supplemented with pyruvate. (A) Real-time analysis of oxygen consumption rates (OCR) and extracellular acidification rates (ECAR) of cultured SGC-7901 cells by perturbing them with small molecule metabolic modulators. Oligomycin (O; 1 µg/ml), carbonyl cyanide p-trifluoromethoxyphenylhydrazone (FCCP; F; 1 µM), oxamate (Ox; 100 mM), and rotenone (R; 1 µM) were injected sequentially at the indicated time points into each well containing SGC-7901 cells after baseline rate measurement. Each data represents mean ± SD. n = 17. (B) Quantitative image analysis of OCR on cultured SGC-7901 cells. Basal, endogenous rate; Oligomycin, ATP-synthase-inhibited rate; FCCP, maximal uncoupled rate; and Rotenone, rotenone-inhibited rate. (C) Basal ECAR and proton production rate from measurements taken in tandem with respiration rates. (D) ATP production rate from oxidative phosphorylation and glycolysis.

We investigated the OCRs and ECAR in cultured SGC-7901 cells using a Seahorse XF-96 extracellular flux analyzer, as described previously [18]. Basal cellular OCR and ECAR were found to be 161.02±29.58 pmol/min per 10×10^3^ cells (initial cell count), and 39.31±4.29 mpH/min per 10×10^3^ cells respectively (Fig. 3A). The ATP-linked respiration (the total basal rate minus the rate with oligomycin, where oligomycin is an inhibitor of ATP synthesis) was 96.15±18.34 pmol/min per 10×10^3^ cells, indicating that ∼60% of cellular oxygen consumption was related to ATP synthesis. Simultaneously ECAR was increased to ∼250% of baseline rates in the presence of maximally effective dose of oligomycin (1 µg/ml), indicating that the cells shifted mitochondrial respiration to glycolysis. Rotenone (1 µM) reduced OCR to 55.78±8.86 pmol/min per 10×10^3^ cells (∼35% of the baseline rates). The rotenone-resistant rate reflects the non-mitochondrial respiration rate, which includes substrate oxidation and cell surface oxygen consumption [19]. Thus, non-mitochondrial respiration accounted for ∼35%, whereas mitochondrial respiration accounted for ∼65% of the total cellular respiration. Thus, in cultured SGC-7901 cells ∼92% (60%/65%) of mitochondrial respiration was coupled to ATP synthesis, and ∼8% of mitochondrial respiration was accounted for by proton leak (Fig. 3B). In the presence of maximally effective dose of FCCP (1 µM, an uncoupling agent that allows maximal electron transport,), a concomitant increase in OCR was observed, and it was increased to 204±30.24 pmol/min per 10×10^3^ cells.

We also assessed the relative contribution of glycolysis and OXPHOS in ATP production rate in SGC-7901 cells. Absolute quantifications of both the glycolytic rate and oligomycin-sensitive oxygen consumption rate were measured in SGC-7901 cells. The extracellular acidification rate is mainly due to lactate and bicarbonate production and, when calibrated as the proton production rate, indicates glycolytic rate [15]. Thus, we used oxamate to inhibit lactate dehydrogenase, which converts pyruvate to lactate during the last step of glycolysis, to calculate the proton production rate (Fig. 3C). There is a one-to-one relationship between the lactate production rate and the ATP production rate from glycolysis. The oligomycin-sensitive oxygen consumption was converted into the ATP production rate using a P/O ratio of 2.3 [20]. The results showed that SGC-7901 cells cultured in DMEM (high glucose) supplemented with 2 mM pyruvate made at least 93% of their ATP using OXPHOS (Fig. 3D).

### Carnosine changed the bioenergetic characterization of SGC-7901 cells

**Figure 4:**
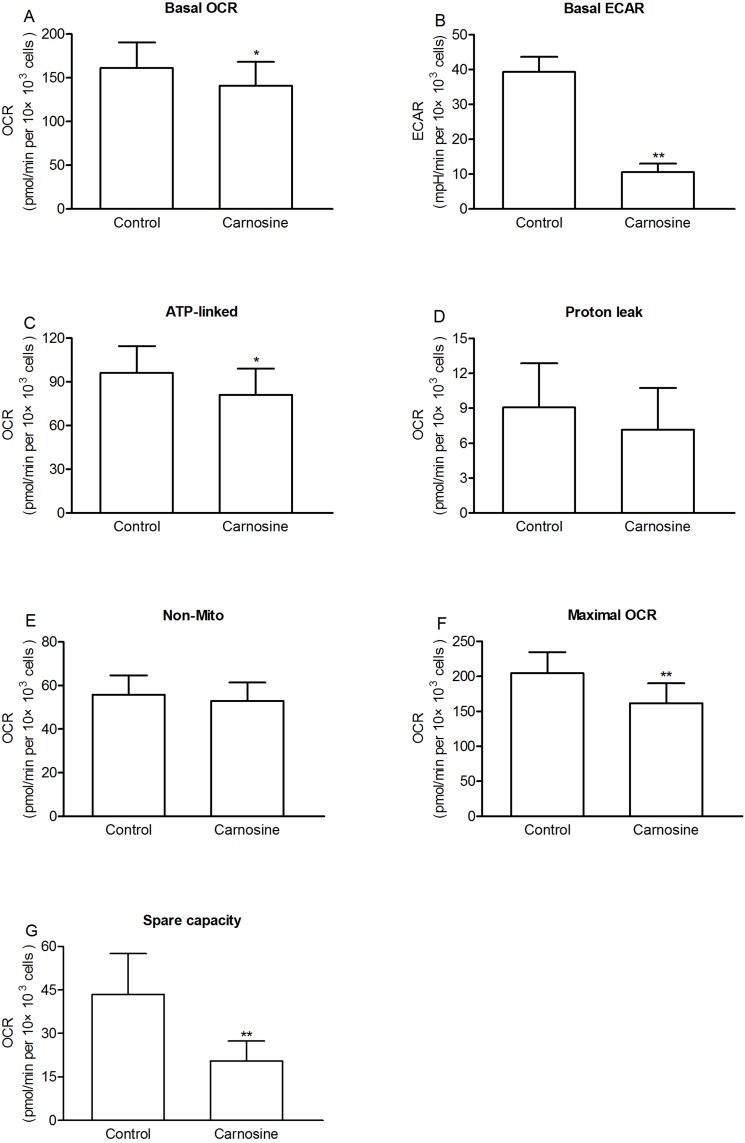
Regulation of mitochondrial respiration by carnosine in cultured SGC-7901 cells. The cells were seeded in specialized microplates and cultured with or without carnosine (20 mM) for 48 h. Cells were then switched to unbuffered DMEM supplemented with 2 mM sodium pyruvate and 20 mM carnosine, and mitochondrial function was assessed using sequential injection of oligomycin, FCCP, and rotenone. (A) Basal OCR, (B) Basal ECAR, (C) ATP-linked OCR, (D) proton leak, (E) non-mitochondrial OCR (Non-Mito), (F) maximal OCR, and (G) spare capacity are shown. Results are means ± SD. n = 6–9. *P<0.05; **P<0.01 vs. control group.

We investigated the effects of carnosine on the oxygen consumption rate and extracellular acidification rate in cultured SGC-7901 cells. The results in Fig. 4 showed that treatment with 20 mM carnosine for 48 h reduced the basal OCR and ECAR to 141.13±27.06 pmol/min per 10×10^3^ cells (∼87% of control), and 11.32±2.49 mpH/min per 10×10^3^ cells (∼27% of control), respectively (Fig. 4A, B). The ATP-linked respiration, proton leak, and non-mitochondrial respiration rate were 81.03±17.97, 7.15±3.6, and 52.96±8.40 pmol/min per 10×10^3^ cells, respectively (Fig. 4C, D, E). Thus in carnosine treated SGC-7901 cells, mitochondrial respiration accounted for ∼62% of the total cellular respiration, and ∼92% (57%/62%) of mitochondrial respiration was coupled to ATP synthesis, and ∼8% of mitochondrial respiration was accounted for by proton leak. Therefore, carnosine treatment decreased the absolute value of ATP-linked respiration, but it did not influence the relative contribution rate of ATP-linked respiration, proton leak, and non-mitochondrial respiration to total cellular respiration. Furthermore, we also found that carnosine treatment markedly reduced the maximal OCR and spare respiratory capacity to 161.60±28.46 pmol/min per 10×10^3^ cells (∼79% of control) and 20.47±6.92 pmol/min per 10×10^3^ cells (∼47% of control) (Fig. 4F, G).

HepG2 and C6 cells were also used to further verify the effects of carnosine on cancer cells energy metabolism. The results showed that 20 mM carnosine treatment for 48 h markedly reduced the basal OCR and ECAR of the HepG2 and C6 cells, respectively. Carnosine addition also reduced ATP-linked respiration in C6 cells, but not in HepG2 cells (Fig. S2). However, 20 mM carnosine markedly reduced the cellular ATP content both in HepG2 and C6 cells, indicating that glycolysis inhibition was involved in carnosine action, at least in HepG2 cells (Fig. S2J).

### Carnosine decreased extracellular lactic acid level in cultured SGC-7901 cells

**Figure 5:**
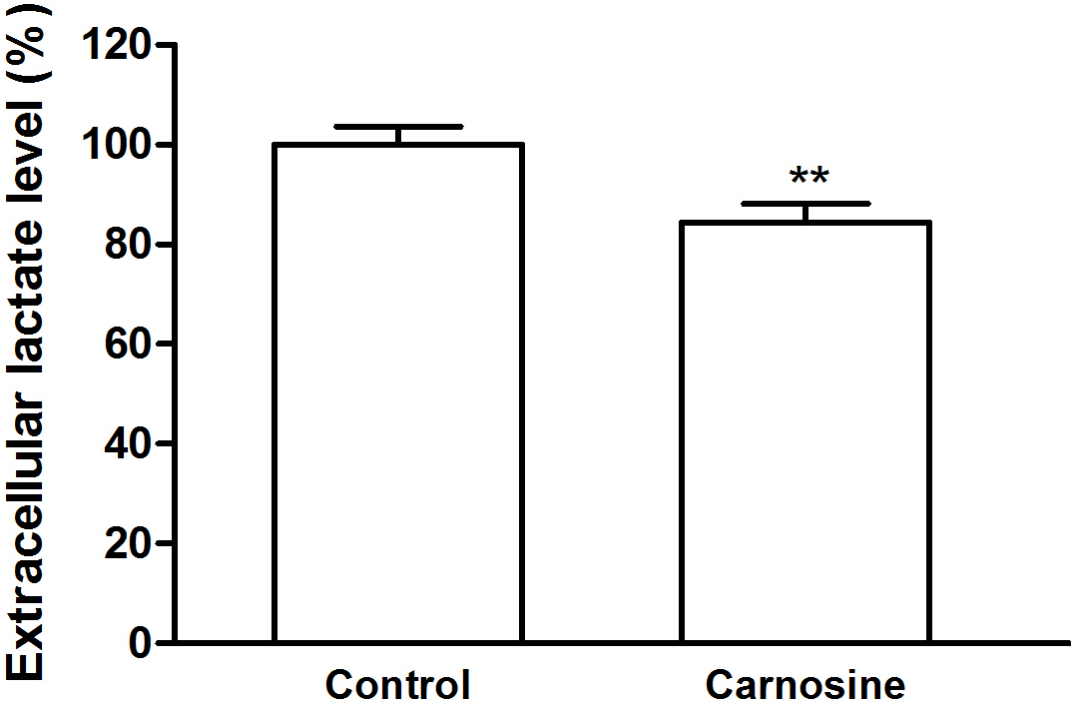
Effect of carnosine on extracellular lactate acid level in cultured SGC-7901 cells. The cells were cultured for 48 h in DMEM with or without carnosine (20 mM). The supernatant was collected for HPLC analysis of lactate acid. Data were expressed as mean ± SD. n = 6. **P<0.01 vs. control group.

Because carnosine is a mobile organic pH buffer, the extracellular acidification rate assayed in the present of carnosine using the Seahorse XF96 Extracellular Flux Analyzer can not reflect the real glycolysis rate. So we also used HPLC to assay the extracellular lactic acid level to verify the effect of carnosine on glycolysis in SGC-7901 cells. Our results showed that carnosine treatment markedly reduced the extracellular lactic acid level to 84% of control (Fig. 5), indicating that carnosine has a potential inhibitive effect on glycolysis in cultured SGC-7901 cells.

### Carnosine changed the relative contribution of glycolysis and OXPHOS to ATP charge in SGC-7901 cells cultured in DMEM lack of pyruvate

**Figure 6:**
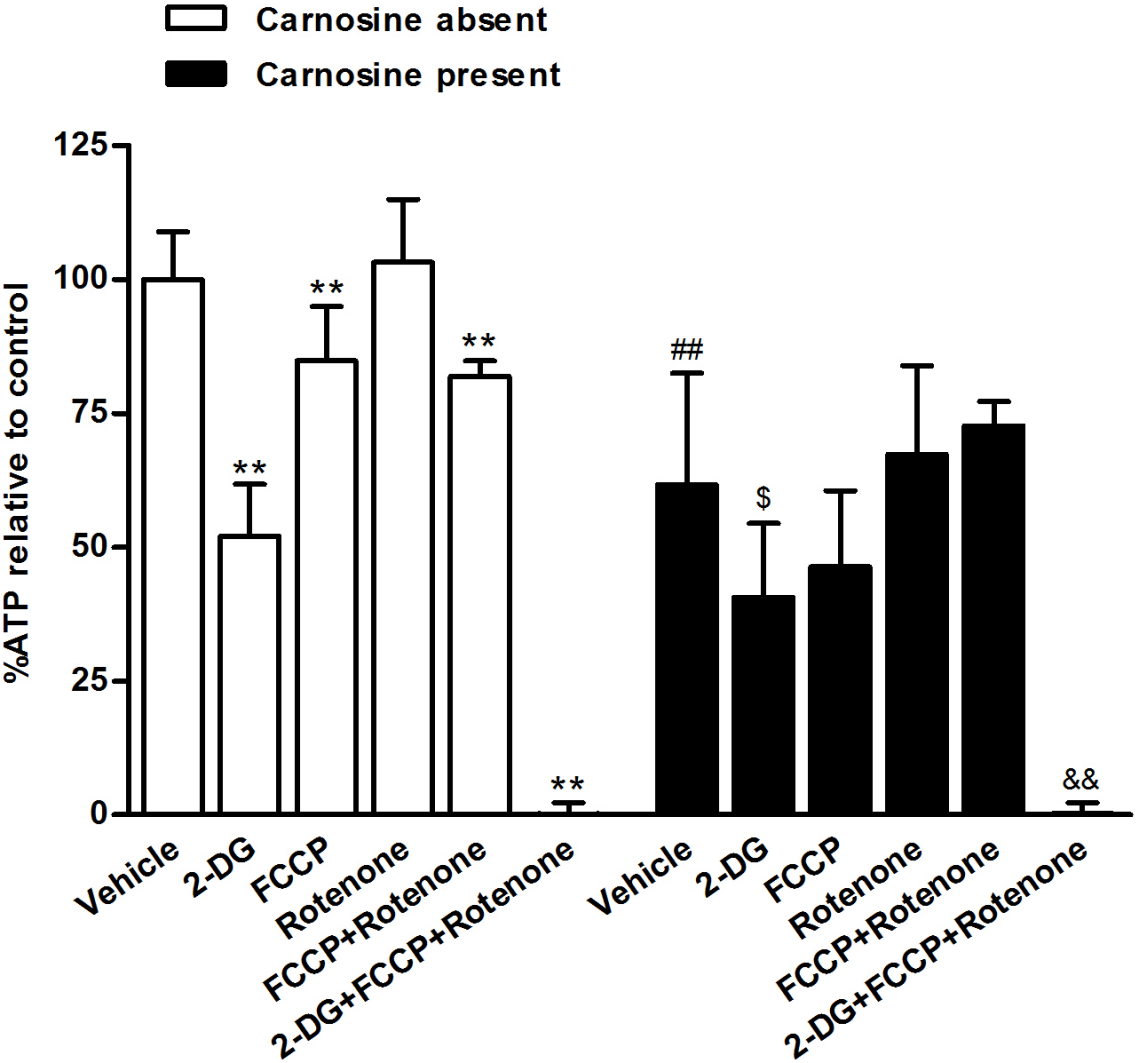
Carnosine changed the relative contribution of glycolysis and OXPHOS to ATP charge in SGC-7901 cells cultured in DMEM free of pyruvate. Intracellular ATP level was measured in response to glycolysis inhibitor 2-deoxyglucose (2-DG, 100 mM), mitochondrial uncoupler FCCP (1 µM), and complex I inhibitor rotenone (1 µM) treatment for 45 min, individually or in combination as shown, in cultured SGC7901 cells with or without carnosine (20 mM) treatment for 48 h. ATP level was expressed as % of control, which was defined as the baseline value in cells exposed only to vehicle. Each data represents mean ± SD. n = 6. **P<0.01 vs. vehicle in carnosine absent group, *^##^P*<0.01 vs. vehicle in carnosine absent group, *^&^P*<0.05, *^&&^P*<0.01 vs. vehicle in carnosine present group.

We also characterized the effect of carnosine on the changes of ATP content and the relative contribution of glycolysis and OXPHOS to ATP charge in SGC-7901 cells cultured in DMEM lack of pyruvate. We measured cellular ATP concentration in cells exposed for 45 min to six conditions: vehicle, the glycolysis inhibitor 2-DG, FCCP, rotenone, FCCP plus rotenone, and 2-DG plus FCCP plus rotenone. We found that carnosine treatment for 48 h significantly reduced ATP content to 62% of control. 2-DG treatment markedly reduced ATP content by 48% and 34% in carnosine absent and carnosine present groups when compared with their own vehicle groups, respectively. FCCP and FCCP plus rotenone each also significantly reduced ATP content by 15% and 18% in carnosine absent group. However, these drugs did not affect ATP content in carnosine present group. Rotenone did not affect ATP content both in carnosine absent or present groups. 2-DG in combination with FCCP and rotenone essentially eliminated ATP production both in these two groups (Fig. 6). Thus 2-DG decreased ATP charge more than FCCP or rotenone did both in carnosine absent and present groups. These data indicate that ATP generation shifts from OXPHOS to glycolysis when mitochondrial function is impaired. ATP charge is not fully maintained after inhibition of either glycolysis or mitochondrial function in carnosine absent group, whereas ATP charge is maintained after inhibition of mitochondrial function in carnosine present group. Thus, carnosine treatment altered the relative contribution of OXPHOS and glycolysis in ATP production rate in SGC-7901 cells.

### Effect of pyruvate on the inhibitive action of carnosine on SGC-7901 cells mitochondrial function

**Figure 7:**
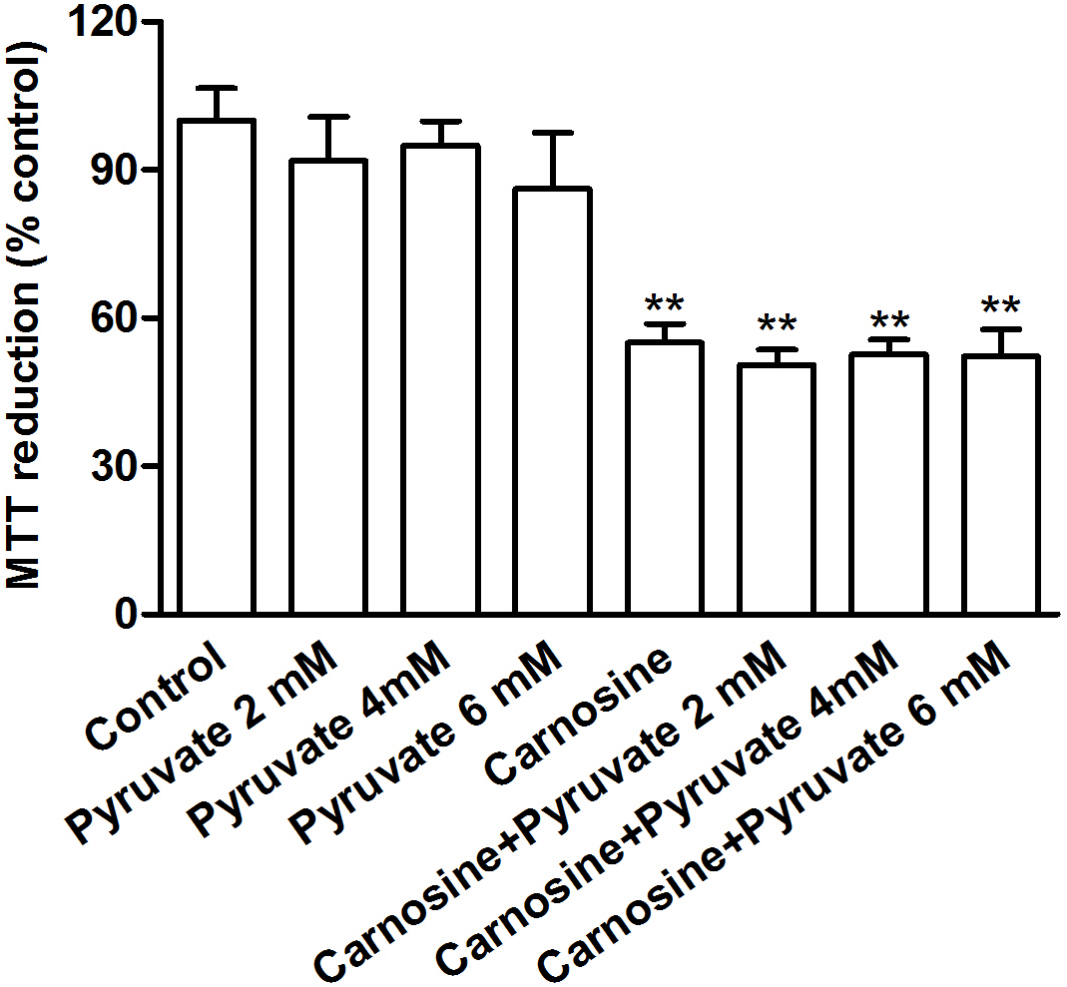
Changes of mitochondrial membrane potential and mtDNA copy number induced by carnosine in SGC-7901 cells. (A) Changes in JC-1 fluorescence with carnosine treatment in cultured SGC-7901 cells. The cells were treated with carnosine (20 mM) for 48 h, and then were stained with JC-1. Red fluorescence indicates a polarized state and green fluorescence indicates a depolarized state. Scale bar: 20 µm. (B) MtDNA copy number in SGC-7901 cells treated with or without carnosine.

To investigate whether increasing the level of pyruvate can reverse the inhibitive effect of carnosine on mitochondrial respiration of SGC-7901 cells, the cells were exposed to different concentrations of pyruvate. As shown in Table. 1, increasing the level of pyruvate could not reverse the inhibitive effect of carnosine on SGC-7901 cells mitochondrial respiration. In addition, the MTT assay also showed that increasing the level of pyruvate could not reverse the carnosine action on SGC-7901 cells mitochondrial metabolism (Fig. 7).

**Table 1:** Mitochondrial function of SGC-7901 cells incubated in different concentrations of pyruvate in the present or absent of carnosine.

	Basal rate	ATP-linked	Mitochondrial	Maximum
Control				
pyruvate 2 mM	161.02±29.58	96.15±18.34	105.23±21.47	204.49±30.24
pyruvate 6 mM	150.07±11.86	92.40±11.73	98.8±11.07	210.81±20.2
Carnosine				
pyruvate 2 mM	141.13±27.06*	81.03±17.97*	88.18±20.16*	161.6±28.46**
pyruvate 6 mM	137.73±22.87	79.42±12.46^##^	89.52±13.81^#^	154.51±30.09^##^

SGC-7901 cells were cultured in DMEM supplemented with 2 or 6 mM pyruvate or this medium supplied with 20 mM carnosine for 48 h. Mitochondrial function was assessed using sequential injection of oligomycin, FCCP and rotenone. Values for basal OCR, ATP-linked OCR, Mitochondrial OCR, and maximal OCR are shown. Data (in pmol/min) are means ± SD with 15–20 replicates. *P<0.05, **P<0.01, compared with control group with 2 mM pyruvate; ^#^P<0.05, ^##^ *P*<0.01, compared with control group with 6 mM pyruvate.

### Effects of carnosine on mitochondrial DNA content and mitochondrial membrane potential in SGC-7901 cells

**Figure 8:**
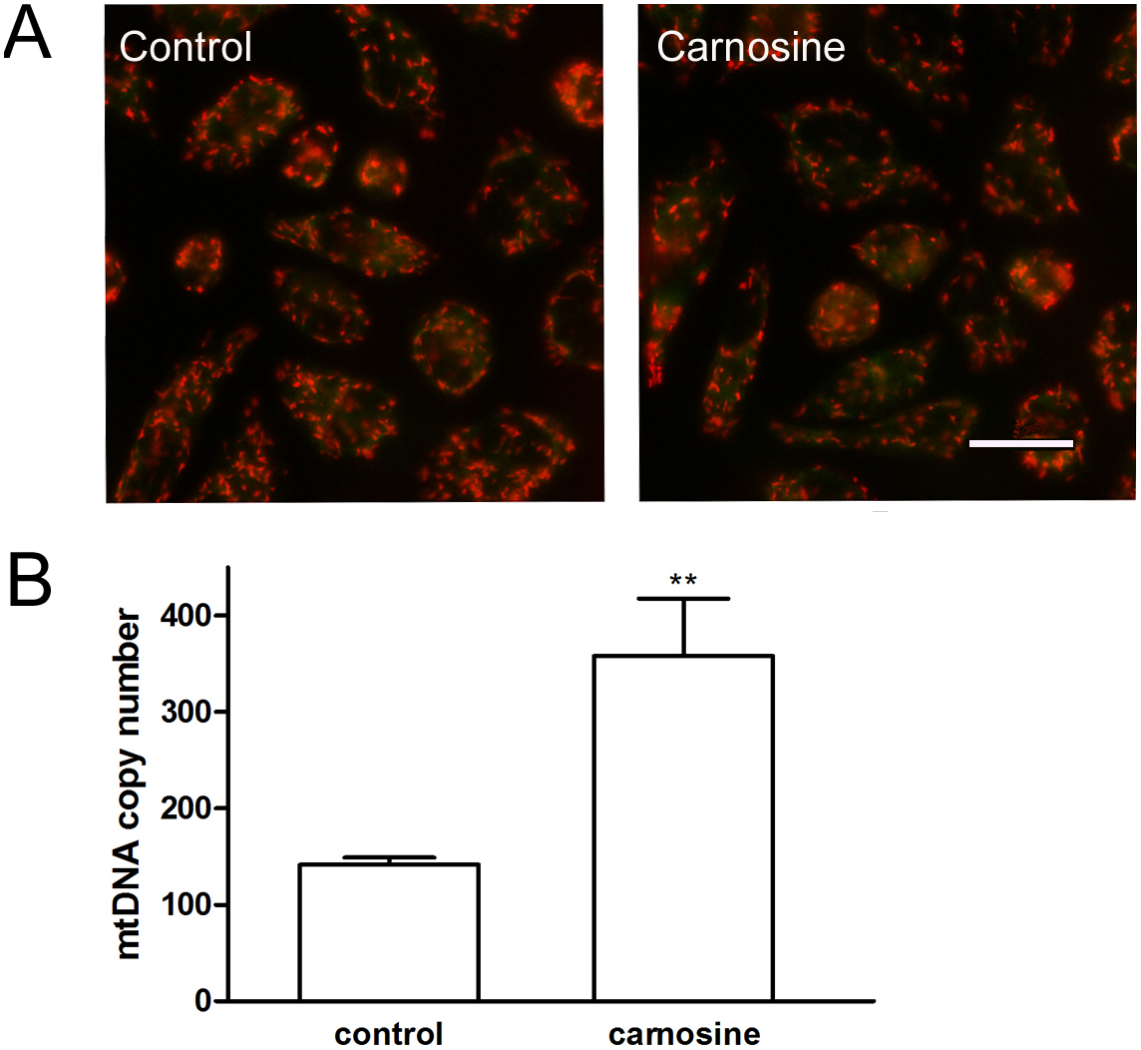
Effect of pyruvate on the inhibitive action of carnosine on SGC-7901 cells mitochondrial function. SGC-7901 cells were cultured in DMEM supplemented with increasing doses of pyruvate (2, 4, 6 mM) in the presence or absence of carnosine (20 mM) for 48 h, and then the cells mitochondrial metabolism activity was measured by MTT reduction assay. Results are mean ± SD. n = 8–16. **P<0.01 vs. control group.

Mitochondrial membrane potential (Δ*Ψ*m) accounts for the majority of the proton-motive force used for driving ATP synthesis and therefore has a significant role in the maximal ATP-generating capacity [21]. Therefore, we also used the lipophilic cationic probe JC-1 to explore whether carnosine suppresses OXPHOS via suppression of the mitochondrial membrane potential (Δ*Ψ*m). The results showed that carnosine treatment for 48 h could not induce an obvious change of Δ*Ψ*m in cultured SGC-7901 cells (Fig. 8A).

To address the apparently decreased mitochondrial respiration induced by carnosine, we also quantified the absolute mitochondrial DNA (mtDNA) number, which is tightly regulated for maintaining cellular energy requirements [22]. To our surprise, the results revealed that compared with control cells, the absolute mtDNA copy numbers of carnosine-treated 7901 cells were markedly increased, and the average absolute mtDNA copy numbers were 141.49±7.42 in the control cells and 360.0±59.84 in the carnosine-treated cells (Fig. 8B).

## Discussion

To our knowledge, this is the first report of the bioenergetic profile of cultured SGC-7901 cells treated with and without carnosine. Our principal findings are as follows. First, carnosine reduced the proliferative capacity of SGC-7901 cells. Second, the cells cultured in DMEM (high glucose) supplemented with 2 mM pyruvate made ∼93% of their ATP using OXPHOS. Third, carnosine exerted its inhibitive effect on SGC-7901 cells proliferation through inhibiting glycolysis, OXPHOS and mitochondrial respiration of the cells, and these actions of carnosine were also found in HepG2 and C6 cells. Thus, carnosine should be considered along with the growing armament of compounds in various stages of drug development that target tumor metabolism, one of the key hallmarks of tumor [23].

Due to its broad spectrum of activity, carnosine may be considerded as a therapeutic factor in the treatment of many diseases. For example, Zinc Carnosine has been used for gastric health and for gut repair [24]. Recently, studies showed transformed cells did not grow in MEM containing high concentrations of carnosine, and carnosine could be administered as a drug in vivo to inhibit the growth of malignant cells [25]. However, it had to be asked whether carnosine can prevent the growth of human gastric tumor cells besides the malignant cells that have been reported previously. In the present study, we found that carnosine is also able to inhibit the growth of cultured SGC-7901 cells in a time- and concentration-related manner, and this effect was not accompanied by apoptosis or necrosis, but may be rather caused by reduced proliferation. However, the detailed mechanisms underlying the inhibitive effect of carnosine on SGC-7901 cells proliferation are still unclear.

It is widely accepted that metabolic changes are one of the hallmarks of cancer. Otto Warburg first described the increased utilization of anaerobic metabolism in the presence of adequate oxygen by cancer cells compared with their normal counterparts: termed the ‘Warburg effect’ [26]. However, recently, it has been pointed out that molecular targeting of OXPHOS may have efficacy for advanced melanoma which have elevated levels of OXPHOS [14]. Thus, different types of tumors on their different development stages have their own metabolic characteristics. Therefore, the therapeutic strategies and mechanisms of different tumors treatment based on energetic metabolism are different. In the present study, we first used a novel extracellular flux technology to assess multiple parameters of mitochondrial function and extracellular acidification rate in parallel with ATP concentration determination to explore the bioenergetic characterization of human gastric cancer SGC-7901 cells. Our Seahorse analysis showed SGC-7901 cells have active mitochondria, and depend on mitochondrial OXPHOS more than glycolysis pathways for generation of ATP in the culture condition with high glucose and pyruvate, suggesting that mitochondrial respiration may be a potential therapeutic target in human gastric cancer.

However, when the cells were cultured in DMEM lack of pyruvate, they depend on glycolysis more than that cultured in DMEM supplied with 2 mM pyruvate, because inhibition of glycolysis by 2-DG leading to a fall of ∼48% cellular ATP content. Moreover, this data also indicate that SGC-7901 cells probably lack plasticity in switching from glycolysis to mitochondrial respiration when glycolytic ATP production is abolished in the culture condition lack of energy substances. On the other hand, Wu *et al*. have reported that there was an effective compensatory upregulation of glycolysis following the administration of oligomycin to block oxidative phosphorylation, and this response was able to sustain ATP level in the human non-small cell carcinoma cell lines H460 and A549 [15]. Our results also showed that SGC-7901 cells have the ability to increase glycolysis when mitochondrial function is blocked by oligomycin. In addition, when the mitochondrial function was suppressed by rotenone, an effective compensatory upregulation of glycolysis was accurred, and this response was able to sustain ATP level in SGC-7901 cells. However, the cells could not upregulate enough glycolysis capacity to sustain ATP level when uncoupling mitochondrial respiration from ATP synthesis induced by FCCP, (Fig. 6). Therefore, SGC-7901 cells possess substantial glycolysis and mitochondrial respartion capacity, and it makes the cells grow under different conditions.

Just recently, it was demonstrated that carnosine reduced proliferation of malignant glioma by an influence on glycolytic and ATP synthesis [27]. In our present study, we also found that treatment with carnosine was capable of decreasing the extracellular acidification rate and lactic acid level, indicating that carnosine has an inhibitive effect on glycolysis in SGC-7901 cells. However, carnosine did not fully, but partially inhibited glycolysis capacity of the cells, because pharmacological inhibition of mitochondrial respiration by rotenone, or uncoupled the mitochondrial proton gradient from ATP production by FCCP, or treated with rotenone and FCCP simultaneously, the cells were still able to upregulate glycolysis to compensate for ATP depletion (Fig. 6). Interestingly, we found that carnosine also possesses a novel role as a regulator of mitochondrial respiration. Carnosine suppressed basal levels of mitochondrial respiration, and this was mainly due to the decreased ATP-linked respiration, indicating that the mitochondrial ATP output decreases in the carnosine-treated SGC-7901 cells. We hypothesized that the mitochondrial respiration capacity of SGC-7901 cells might be impaired after carnosine treatment and therefore is less able to upregulate mitochondrial respiration to compensate for ATP depletion caused by the pharmacological inhibition of glycolysis. To test this hypothesis, we determined the mitochondrial respiration capacity of the cells using FCCP. Indeed, we found that the ability of SGC-7901 cells to increase their respiration by FCCP was highly compromised (Fig. 4F, G). Thus, all the data suggest that carnosine is a regulator of energy metabolism both in the anaerobic and aerobic pathways in SGC-7901 cells. Furthermore, the inhibitive action of carnosine on cell proliferation was also found in HepG2 and C6 cells, and mitochondrial respiration and glycolysis inhibition induced by carnosine was also found in HepG2 and C6 cells, indicating that carnosine is a nonspecific anti-tumor agent.

So far, the mechanisms of regulation the energy metabolism by carnosine in SGC-7901 cells are unknown. Recently, it has been reported that the terminal amino group of carnosine can react strongly with aldehyde and keto groups of sugars. Thus, it was proposed that carnosine depletes glycolysis intermediates, reduces production of pyruvate by glycolysis, and therefore reduces the generation of ATP by this anaerobic pathway and further in turn limits the production of ATP by the TCA cycle [25]. Here, we found that adding exogenous pyruvate to the culture media of SGC-7901 cells which express monocarboxylate transporters MCT4 and MCT1 that can transport pyruvate did not affected the carnosine action on the cells mitochondrial respiration. In addition, carnosine also could decrease ATP charge in SGC-7901 cells cultured in DMEM lack of pyruvate (Fig. 6). Thus, our data suggested that pyruvate may not involve in the carnosine action on the cellular energy metabolism, at least in the cultured SGC-7901 cells.

The cells treated with carnosine had a similar mitochondrial membrane potential with control cells, reflecting mitochondrial membrane potential is not involved in the inhibitive action of carnosine on mitochondrial respiration and OXPHOS of the cells. Interestingly, in contrast to decreased mitochondrial respiration and OXPHOS, the mtDNA copy number was markedly increased by carnosine in SGC-7901 cells. MtDNA encodes 2 ribosomal and 22 transfer RNAs, and 13 subunits of the electron transfer chain (ETC), which is the major generator of cellular ATP through OXPHOS) [28], [29]. Thus, regulation of mtDNA copy number is essential for maintaining cellular energy requirements [22]. However, alterations in mtDNA copy number have been observed in a variety of human cancers. Decreased mtDNA copy number in some cancers may be due to the increased glycolysis, whereas in some other cancers the mtDNA copy number is increased, and it is probably a compensatory action upon the disorders occurring in the mitochondrial respiration chain and ATP generation [22], [30]–[32]. Thus, the increased mtDNA copy number in SGC-7901 cells may be an effective compensatory upregulation of mitochondrial respiration and OXPHOS following the administration of carnosine to block mitochondrial function and glycolysis. Additional studies are certainly needed to determine the detail mechanisms underlying the carnosine action on the SGC-7901 cells energy metabolism.

In conclusion, our results demonstrate that mitochondria plays the primary role in maintaining energy homeostasis in SGC-7901 cells cultured in DMEM supplemented with pyruvate, while glycolysis makes much more contribution in the culture condition lack of pyruvate. The present study highlights a novel role of carnosine as a regulator of SGC-7901 cells energy metabolism both in the anaerobic and aerobic pathways, and describes an alternative mechanism of action for energy metabolism regulators which may give renewed impetus for their development as anti-tumor agents.

## Supporting Information

Figure S1 Effects of carnosine on HepG2 and C6 cell viability and proliferation. (A) Cells were pre-treated with 5 and 20 mM carnosine for 48 h, and then the cell viability was assayed using the MTT reduction assay. Results were expressed as percentage of control, and were showed mean ± SD. n = 10–12. (B) HepG2 and C6 cells were treated with 20 mM carnosine and the total cell number was calculated after carnosine treatment for 2, 3, 4, 5, 6 days using cell counting plate. Data were expressed as mean ± SD. n = 6. *P<0.05, **P<0.01 vs. control in HepG2 cells group; ^##^ *P*<0.01 vs. control in C6 cells group.
(TIF)

Figure S2 Regulation of oxygen consumption rates (OCRs), extracellular acidification rates (ECAR) and cellular ATP content by carnosine in cultured HepG2 and C6 cells. The cells were seeded in specialized microplates and cultured with and without carnosine (20 mM) for 48 h. Cells were then switched to unbuffered DMEM supplemented with 2 mM sodium pyruvate and 20 mM carnosine. (A) Real-time analysis of OCR and (B) ECAR of cultured HepG2 and C6 cells by perturbing them with small molecule metabolic modulators. Oligomycin (O; 1 µg/ml), FCCP (F; 1 µM), oxamate (Ox; 100 mM), and rotenone (R; 1 µM) were injected sequentially at the indicated time points into each well containing HepG2 or C6 cells after baseline rate measurement. (C) Basal OCR, (D) Basal ECAR, (E) ATP-linked OCR, (F) proton leak, (G) non-mitochondrial OCR (Non-Mito), (H) maximal OCR, and (I) spare capacity are shown. (J) Effects of carnosine on the cellular ATP content in HepG2 and C6 cells. ATP level was expressed as % of control. Results are means ± SD. n = 4–6. *P<0.05; **P<0.01 vs. control in HepG2 cells group; ^##^ *P*<0.01 vs. control in C6 cells group.
(TIF)

Table S1 Flow cytometric analysis of cell necrosis or apoptosis induced by carnosine in HepG2 and C6 cells.
(DOC)
