# Supplementary figures and images for: Carnosine Inhibits the Proliferation of Human Gastric Cancer SGC-7901 Cells through Both of the Mitochondrial Respiration and Glycolysis Pathways
Source: PLoS One. 2014 Aug 12;9(8):e104632. doi: 10.1371/journal.pone.0104632 (PMC4130552; doi:10.1371/journal.pone.0104632)

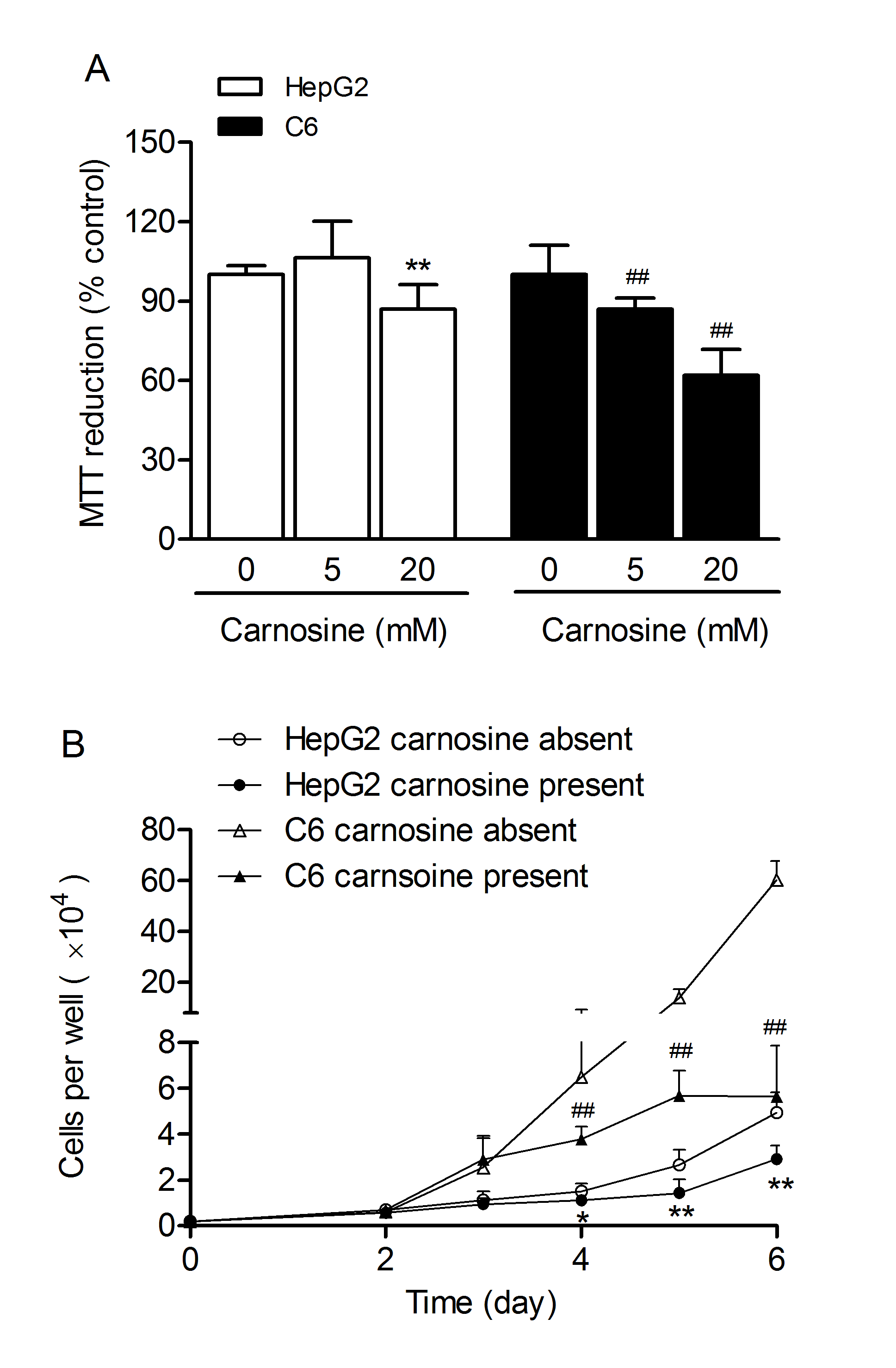

Supplement: Figure S1 — Effects of carnosine on HepG2 and C6 cell viability and proliferation. (A) Cells were pre-treated with 5 and 20 mM carnosine for 48 h, and then the cell viability was assayed using the MTT reduction assay. Results were expressed as percentage of control, and were showed mean ± SD. n = 10–12. (B) HepG2 and C6 cells were treated with 20 mM carnosine and the total cell number was calculated after carnosine treatment for 2, 3, 4, 5, 6 days using cell counting plate. Data were expressed as mean ± SD. n = 6. *P<0.05, **P<0.01 vs. control in HepG2 cells group; ## P<0.01 vs. control in C6 cells group. (TIF) [file pone.0104632.s001.tif]

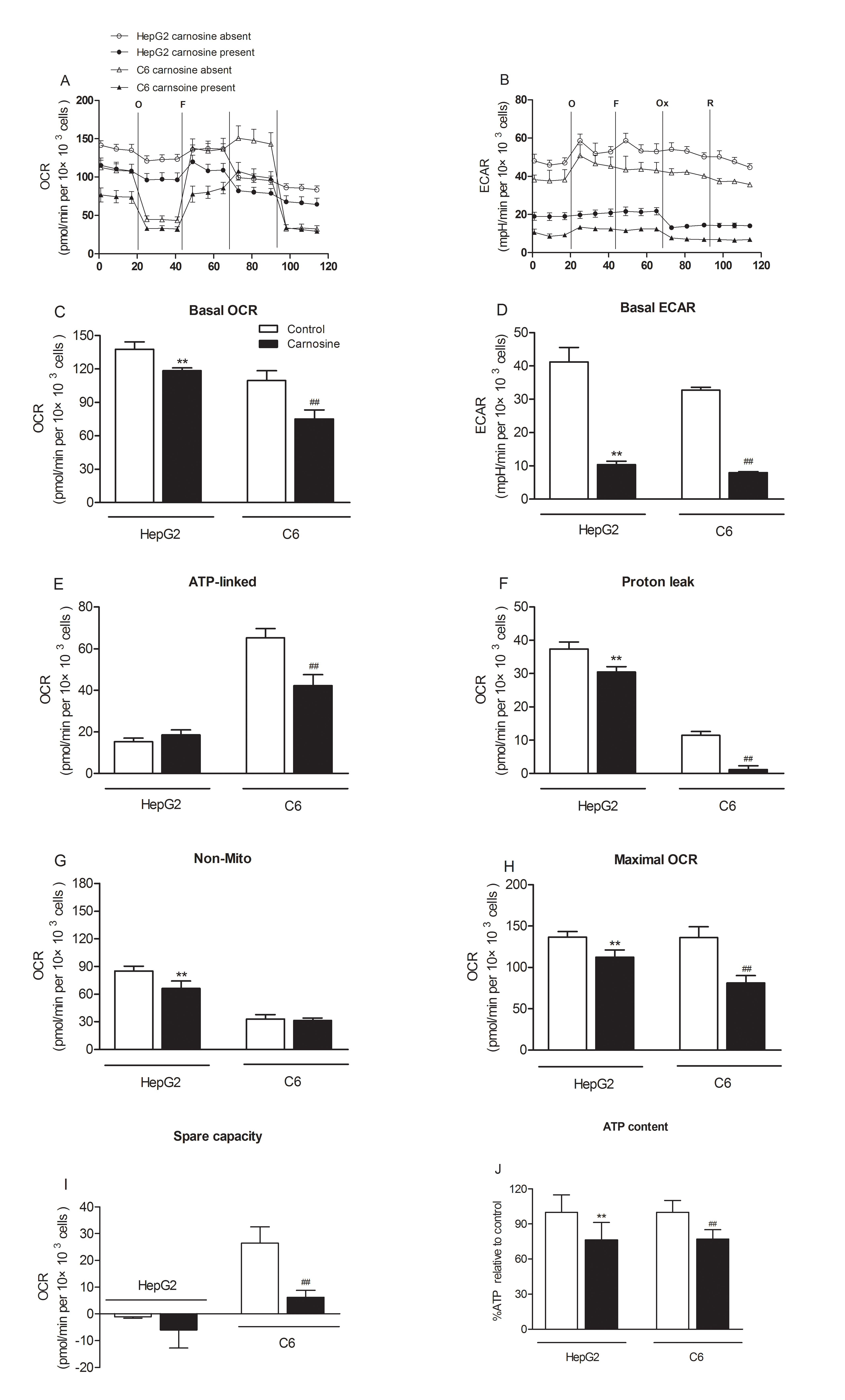

Supplement: Figure S2 — Regulation of oxygen consumption rates (OCRs), extracellular acidification rates (ECAR) and cellular ATP content by carnosine in cultured HepG2 and C6 cells. The cells were seeded in specialized microplates and cultured with and without carnosine (20 mM) for 48 h. Cells were then switched to unbuffered DMEM supplemented with 2 mM sodium pyruvate and 20 mM carnosine. (A) Real-time analysis of OCR and (B) ECAR of cultured HepG2 and C6 cells by perturbing them with small molecule metabolic modulators. Oligomycin (O; 1 µg/ml), FCCP (F; 1 µM), oxamate (Ox; 100 mM), and rotenone (R; 1 µM) were injected sequentially at the indicated time points into each well containing HepG2 or C6 cells after baseline rate measurement. (C) Basal OCR, (D) Basal ECAR, (E) ATP-linked OCR, (F) proton leak, (G) non-mitochondrial OCR (Non-Mito), (H) maximal OCR, and (I) spare capacity are shown. (J) Effects of carnosine on the cellular ATP content in HepG2 and C6 cells. ATP level was expressed as % of control. Results are means ± SD. n = 4–6. *P<0.05; **P<0.01 vs. control in HepG2 cells group; ## P<0.01 vs. control in C6 cells group. (TIF) [file pone.0104632.s002.tif]
