# Supplementary material for: Carnosine Inhibits the Proliferation of Human Gastric Cancer SGC-7901 Cells through Both of the Mitochondrial Respiration and Glycolysis Pathways
Source: PLoS One. 2014 Aug 12;9(8):e104632. doi: 10.1371/journal.pone.0104632 (PMC4130552; doi:10.1371/journal.pone.0104632)
Supplement: Table S1 — Flow cytometric analysis of cell necrosis or apoptosis induced by carnosine in HepG2 and C6 cells. (DOC) [file pone.0104632.s003.doc]

**Table S1** Flow cytometric analysis of cell necrosis or apoptosis induced by carnosine in HepG2 and C6 cells

| Treatment | Annexin V^-^/PI^+^ (%) (necrosis) | Annexin V^+^/PI^+/-^ (%) (early/late apoptosis) |
| --- | --- | --- |
| HepG2 |  |  |
| Control | 0.68 ± 0.17 | 3.6 ± 0.47 |
| Carnosine | 0.70 ± 0.14 | 3.5 ± 1.04 |
| C6 |  |  |
| Control | 1.25± 0.62 | 4.03 ± 1.16 |
| Carnosine | 1.3 ± 0.37 | 4.2 ± 1.26 |

HepG2 and C6 cells were treated with carnosine (20 mM) for 48 h. The cell death was determined by PI and annexin V-FITC staining followed by flow cytometry. Each value is expressed as mean ± SD, n = 4.
